# Insights into the Maternal Ancestry of Côte d’Ivoire Honeybees Using the Intergenic Region *COI*-*COII*

**DOI:** 10.3390/insects10040090

**Published:** 2019-03-29

**Authors:** Krouholé Abdoul Salam Coulibaly, Muhammad Zeeshan Majeed, Chao Chen, Kolo YEO, Wei Shi, Chun-Sen Ma

**Affiliations:** 1 Climate Change Biology Research Group, State Key Laboratory for Biology of Plant Diseases and Insect Pests, Institute of Plant Protection, Chinese Academy of Agricultural Sciences, Beijing 100193, China; ckcheick@yahoo.fr (K.A.S.C.); shani2000_uaf@yahoo.com (M.Z.M.); 2 Institute of Apicultural Research, Chinese Academy of Agricultural Sciences, Beijing 100093, China; chao_chen@outlook.com; 3 Department of Entomology, College of Agriculture, University of Sargodha, Sargodha 40100, Pakistan; 4 Station d’écologie de Lamto, Université Nangui Abrogoua, Abidjan BP 28, Côte d’Ivoire; koloyeo@yahoo.fr

**Keywords:** *Apis mellifera*, genetic diversity, haplotypes, Côte d’Ivoire, population structure

## Abstract

Honeybee populations in Côte d’Ivoire have been previously identified as belonging to one subspecies, *Apis mellifera scutellata*, but other studies have since reported a mixed population consisting of *A. m. adansonii* and *A. m. jemenitica.* The population structure and the geographic distribution of honeybees in Côte d’Ivoire remain unclear. This study aimed to profile the population structure of honeybees and their biogeography in Côte d’Ivoire. A total of 33 honeybee colonies were sampled from 15 localities to investigate the maternal ancestry of indigenous honeybee populations using the DraI *COI*-*COII* mtDNA test. The results revealed that the honeybee population in Côte d’Ivoire is composed of African haplotypes, all belonging to the A_I_ sublineage. Haplotypes A_1_ and A_4_ were recorded with five new sequence variants, including three types of haplotype A_1_ and two types of haplotype A_4_. The A_1e_ variant was the most frequent in the *A. m. adansonii* distributional area. The distribution of the haplotype variants was correlated with the climate pattern in Côte d’Ivoire. This is the first study in Côte d’Ivoire that gives insights into the biogeography and mitotype structure of the local honeybee populations.

## 1. Introduction

Based on morphometrics and multivariate analyses [1], the honeybee species *Apis mellifera* L. has been split into four lineages. Lineage A is spread from the north to the south of Africa, lineages M and C are distributed in Europe and lineage O in the Middle East [1,2,3]. Recently, the DraI mitochondrial DNA test was used in the identification of maternal honeybee populations [3,4,5,6,7,8,9]. Using this method, four mitochondrial DNA (mtDNA) lineages have been identified, including lineages A, M, C, and Y [2,7,10]. Honeybees from the M and C lineages have been more extensively studied for their taxonomy, biodiversity, and geographic distribution than their counterparts, the A and Y lineages. The genetic diversity of honeybees in the African continent is still understudied [3,11,12], making genetic conservation programs difficult in Africa [12]. To gain further insights into the genetic diversity of indigenous honeybee populations, more studies are needed to improve the availability of reference data in different African regions. This information could contribute towards monitoring the endemic populations, improving honeybee services, and designing strategies for ecological conservation of the local population. Several studies have been done to discriminate the honeybee population in Africa [1,3,13,14,15,16,17,18,19,20,21,22]. Using morphometric traits, eleven *A. mellifera* L. subspecies have been taxonomically recognized in the African continent, i.e., *A. m. lamarckii* [14], *A. m. jemenitica* [15], *A. m. intermissa* [16], *A. m. sahariensis* [17], *A. m. unicolor* [18], *A. m. scutellata* [19], *A. m. capensis* [20], *A. m. adansonii* [18], *A. m. monticola*, *A. m. litorea* [21], and *A. m. sinensis* [22].

In West Africa, particularly in Côte d’Ivoire, the biodiversity of honeybee populations has been studied with sample sizes that are low for the size of the region (322,462 km^2^). Classification of local honeybee populations in Côte d’Ivoire has, therefore, been controversial. Based on the morphometric characterization, the honeybee populations in Côte d’Ivoire were initially claimed to be *A. m. scutellata* [19]. Later, Ruttner [1] recognized these populations as belonging to *A. m. adansonii* in the tropical dry and subequatorial climates. Similarly, according to the morphometric study carried out by Radloff et al. [13], honeybee populations from Côte d’Ivoire were identified as *A. m. adansonii* in the tropical dry and tropical humid climates, with some *A. m. jemenitica* and *A. m. adansonii* hybrid populations.

Mitochondrial DNA variation in the cytochrome oxidase subunit I-II (*COI*-*COII*) intergenic region has been used to distinguish lineages and to refine the classification of *A. mellifera* L. [2,23,24,25,26]. Mitochondrial DNA markers were employed to demonstrate that the honeybee populations from Côte d’Ivoire belong to lineage A [3,27]. However, lineage A consists of four sublineages, namely, A_I_ (haplotype A_1_–A_4_, A_6_, A_12_, A_13_, A_19_, A_24_–A_27_, etc.), A_II_ (haplotype A_8_, A_10’_ A_9_, etc.) [3], A_III_ (haplotype A_20_, A_11_, A_14_, A_30_, A_33_, etc.) [28], and Z (haplotype Z_1_–Z_4_, Z_7_) [29,30]. These sublineages are usually differentiated by the presence or absence of an additional DraI site (TTTAAA) and a deletion at the 3’ end of the P element [2,3]. Two forms of the P element, P_0_ and P_1_, are typical of lineage A. The P_1_ form is characterized by a 15-bp deletion at the 3’ end of the P element, whereas P_0_ does not exhibit any large deletion. The P_0_ form is carried by sublineages A_I_, A_II_, and Z [3,29,30], whereas P_1_ is carried by sublineage A_III_ [28]. Sublineage A_II_ is differentiated from sublineage A_I_ by the absence of the DraI site at the 5’ end of the first Q element, whereas sublineage Z has an additional DraI site in the middle of the first Q element. The classification into sublineages and the haplotype geographical distribution patterns of the honeybee populations of Côte d’Ivoire are yet to be clarified. Getting an accurate view of the genetic diversity could allow targeting of the sensitive honeybee populations and conservation of their biodiversity.

In this study, we provide for the first time the matriline structure and biogeographic distribution of the honeybee populations in Côte d’Ivoire using the highly polymorphic intergenic *COI*-*COII* region of the mtDNA.

## 2. Materials and Methods 

### 2.1. Study Area

**Figure 1:**
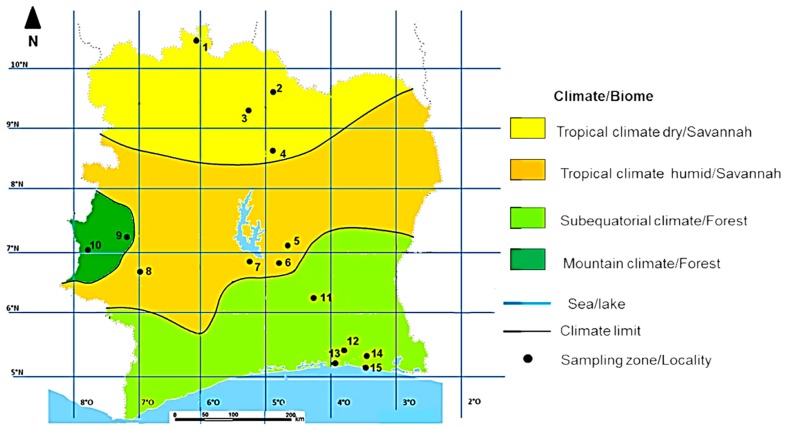
Map of honeybee sampling sites along with different climate and biome types in Côte d’Ivoire. Each sampling locality is presented with a black dot. The 15 sampling localities were spread over the 4 climate types (tropical dry and humid climates, subequatorial dry, and mountain climates) and the 2 biome types (Savannah and Forest). The numbers in the map represent a site with correspondence names and coordinates shown in Table 1.

The study was conducted in Côte d’Ivoire, a West African country geographically located at 5°18′34″ N and 4°00′45″ W, between the Sahara Desert and the Atlantic Ocean. The country has four climate types from the north to the south (Figure 1), i.e., tropical dry climate, tropical humid climate, subequatorial climate, and a mountain climate that is found on the western side (Table 1).

### 2.2. Collection of Honeybees

Between May and July, 2015, sixty adult worker honeybees were collected from each of 33 colonies from 15 localities in Côte d’Ivoire (Table 1). Ten colonies were sampled from the tropical dry, tropical humid, and subequatorial climates each, and three colonies were sampled from the mountain climate (Table 1). Samples were taken from feral swarms collected by the traditional beekeepers in the countryside. All samples were preserved in 90% ethanol and were transferred under cool conditions to the Institute of Apicultural Research (IAR), Chinese Academy of Agricultural Sciences, Beijing, China, for their molecular characterization. The low sample size collected in Côte d’Ivoire was mainly due to the quasi absence of beekeeping in several regions. Moreover, in the selected regions, beekeepers (only 18) held less than five hives. 

### 2.3. DNA Extraction, Sequencing, and DraI Test

One worker honeybee from each sampled colony was subjected to DNA extraction. The DNA was extracted from the individual honeybee thoraces by using an E.Z.N.A^®^ Tissue DNA Kit (Omega Bio-Tek, Doraville, GA, USA) according to the manufacturer’s instructions. 

PCR amplification of the *COI*-*COII* intergenic region was carried out according to a protocol detailed previously [4]. The size of the amplified DNA amplicon was determined by running 10 μL of the PCR-amplified products for 20 min on 1.0% agarose gel using gel electrophoresis. After gel migration, it was visualized and photographed using a UV-equipped gel documentation system (Bio-Rad laboratories, 6000; Biorad, Hercules, CA, USA). About 20 μL of PCR products from each sample were sent to Sangon Biotech (Beijing, China) for purification and direct Sanger sequencing in both directions. To confirm the result quality of the sequenced DNA, we repeated the same analysis independently with two more individuals from each colony, yielding a total of three sequences per colony. 

To conduct the restriction fragment length polymorphism (RFLP) analysis, we used DraI (Boehringer Manheim) to digest 500 ng of the PCR products from each colony individually. The digested products were then run on 2.0% Metaphor agarose gels prepared in 1 × TBE at 10 V/cm to carry out the electrophoretic analysis. The nucleotide bands were visualized under a UV transilluminator [31]. 

### 2.4. Data Analysis

Before data analysis, we used the CodonCode aligner (www.codoncode.com) to clean the sequences. Then, the mtDNA sequences were aligned using MEGA 5.04 software [32]. Comparisons of the sequences were conducted with the Basic Local Alignment Search Tool (BLAST^®^) by searching the most relevant DNA sequences available on the GenBank^®^ web portal (http://blast.ncbi.nlm.nih.gov/Blast.cgi). Newly described sequences were submitted to the GenBank NCBI database under the accession numbers MF984182, MF984186, MH152663, MH152664, and MH152665. Similarity among the *COI*-*COII* haplotypes was investigated using the PopART version 1.6 (Population Analysis with Reticulate Trees) software (http://popart.otago.ac.nz). The classical phylogenetic tree, including lineages M, C, and Y, and sub-lineages A_I_, A_II_, A_III_, Z, was built. We trimmed all nucleotide sequences to the same length. Then, the data was aligned on the online service of www.ebi.ac.uk. Using the software Jalview version 2.10.3 [33], the parsimony tree of mtDNA *COI*-*COII* was generated by average distance with the percentage identity method (PID). 

## 3. Results

Honeybee populations from Côte d’Ivoire were composed of two mtDNA sequences, P_0_Q and P_0_QQ, corresponding to the sequence lengths of 545 and 737 bp, respectively. P_0_Q and P_0_QQ types were described by the haplotype A_1_ [2] and A_4_ respectively. While P_0_Q was the most recorded with an overall presence in 31 out of 33 sampled honeybee colonies, P_0_QQ was found in 2 out of 33. Among the 33 colonies examined, we recorded four variants of haplotype A_1_, i.e., A_1e_ [34], A_1s_, A_1q_, and A_1r_, and two variants of haplotype A_4_, i.e., A_4o_ and A_4r_. In this study, the haplotype variants A_1s_, A_1q_, A_1r_, A_4o_, and A_4r_ were newly described. The sequence A_1e_ was the most dominant (23 out of the 33) in the samples. 

**Figure 2:**
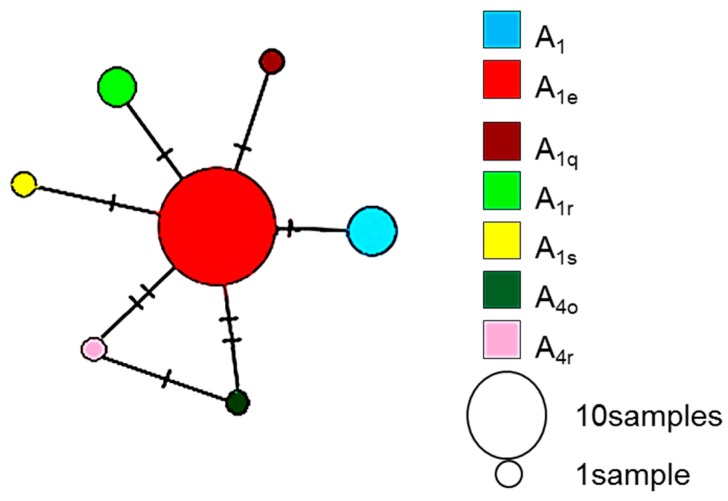
Haplotype network displaying the relationship between the mtDNA sequences of *Apis mellifera* L. samples collected from different localities in Cote d’Ivoire. Circle size represents the number of haplotype copies recorded in the dataset. Each hatch mark represents a nucleotide change.

**Figure 3:**
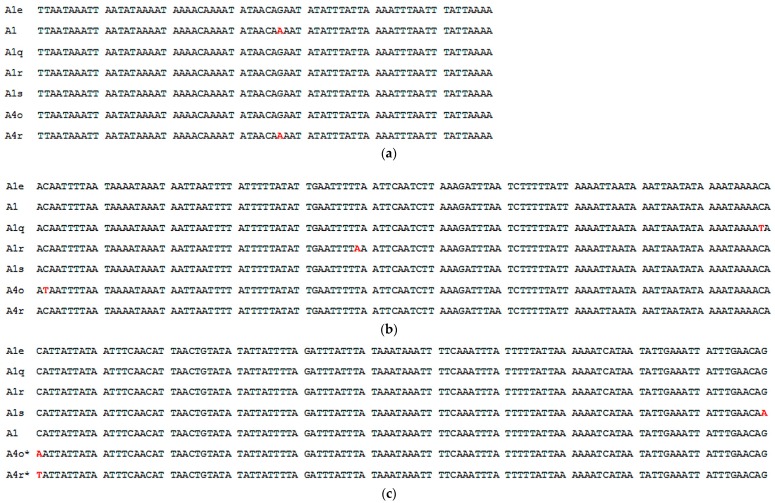
Pattern of P_0_ sequences section from 1 to 68bp (**a**) and Q sequences section of the *COI*–*COII* intergenic region of A_1_, A_1e_, A_1s_, A_1q_, A_1r_, A_4o_, A_4r_ from 69 to 218 bp (**b**) and from 339 to 468 bp (**c**). Asterisk (*) mark indicates 2nd Q section of A_4o_ and A_4r_ from 459 to 589 bp. Letters in red correspond to mutation sites.

The sequences A_1_, A_1e_, A_1s_, A_1q_, A_1r_, A_4o_, and A_4r_ comprised one haplogroup by network analysis (Figure 2). The sequence A_1e_ represents the internal component of the haplogroup. The sequences A_1_, A_1s_, A_1q_, and A_1r_ diverged from the dominant sequence A_1e_ by only one base (Figure 3) and the sequences A_4o_ and A_4r_ were separated from A_1e_ by two bases. 

**Figure 4:**
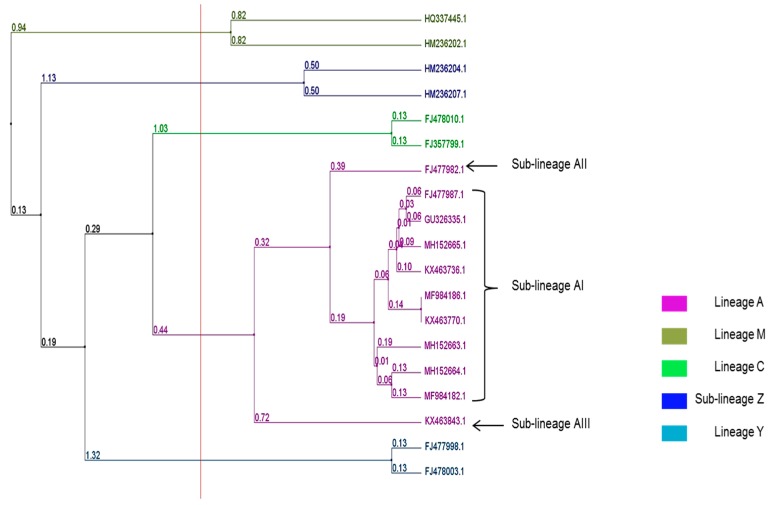
Phylogenetic tree of the mtDNA *COI*-*COII* sequence A_1s_ (MH152665), A_1q_ (MF984182), A_1r_ (MF984186), A_4o_ (MH152663), A_4r_ (MH152664), A_1_ (KX463736), A_1e_ (GU326335) of honeybee populations in Côte d’Ivoire. Additionally, the observed mtDNA sequence recorded in the lineage Y (FJ478003 = Y_2d_, FJ477998 = Y_1a_), M (HQ337445 = M_12_, HM236202 = M_63_), C (FJ478010 = C_1_, FJ357799 = C_1b_), and A with the sublineages A_I_ (KX463770 = A_6_, FJ477987 = A_4_), A_II_ (FJ477982 = A_9_), A_III_ (KX463843 = A_20_) and Z (HM236204 = Z_1_, HM236207 = Z_3_). Distances are indicated near the node.

Our results clearly show that Côte d’Ivoire honeybee colonies belong to the A lineage and the A_I_ sublineage. The P_0_Q sequence pattern of A_1e_, A_1s_, A_1q_, and A_1r_ and the P_0_QQ sequence pattern of A_4o_ and A_4r_ from the collected data were genetically close to the sequence pattern of the honeybee *A. m. scutellata* mtDNA of haplotype A_4_ recorded in South Africa [5] and *A. m. adansonii* matrilines of haplotype A_1_ recorded in Zambia [3] (Figure 4). 

**Figure 5:**
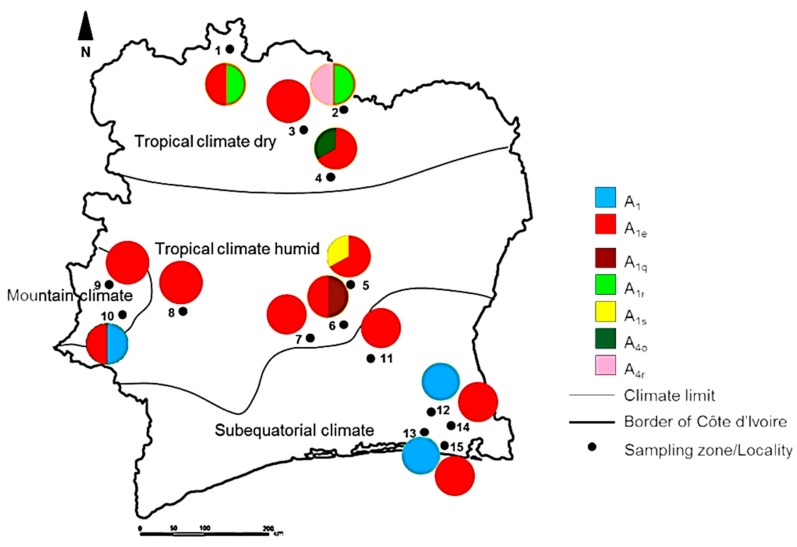
Map of Côte d’Ivoire showing the distribution of *COI*-*COII* according to the sample locations (1 to 15). Pie-charts indicate the frequencies of sequence at each location based on our collected samples.

The sequence A_1e_ was distributed across all climatic types (i.e., dry tropical, humid tropical, mountain, and subequatorial climates) in both savannah and forest biotypes (Figure 5) with a frequency of six in the dry tropical climate, eight in the humid tropical climate, one in the mountain climate, and eight in the subequatorial climate. We found that A_1r_ was only distributed in the dry tropical climate in the savannah, and A_1q_ and A_1s_ were recorded only in the humid tropical climate, in the savannah biome. Haplotype A_1_ was recorded in the subequatorial climate in the forest biotype, while the haplotype A_4_ and its variants A_4o_ and A_4r_ were predominant in the northern part of the country in the tropical dry climate of the savannah biotype. With the exception of A_1e_, there was a clear co-segregation of haplotype variants and climatic types.

## 4. Discussion

Among the sequences of haplotype A_1_ and A_4_ (i.e., A_1e_, A_1s_, A_1q_, A_1r_, A_4o_, and A_4r_) recorded in this study from the Côte d’Ivoire, three variants of haplotype A_1_, namely A_1s_, A_1q_, and A_1r_, and two variants of haplotype A_4_, namely A_4o_ and A_4r_, were not yet recorded and are described in this study. The haplotype A_1_ and its variant A_1e_ were previously reported by Chávez-Galarza et al. [2] from Iberia and by Szalanski and Magnus [34] from Washington and Iron Counties, Utah, USA, in NCBI GenBank using mtDNA markers. All of them belonged to the evolutionary lineage A, particularly to the A_I_ sublineage, indicating that honeybees from Côte d’Ivoire belong exclusively to the African lineage [3,13]. The hybridization with *A. m. jemenitica* from lineage Y, as reported earlier, may occur, between *A. m. adansonii* queen and *A. m. jemenitica* drone. Therefore, haplotypes of Y lineage ancestry (*A. m. jemenitica*) could not be found in our samples. The absence of Y lineage in our study does not mean that they are not present at all in Côte d’Ivoire. The rarity of *A. m. jemenitica* could be due to an inadequate environment, mainly due to low altitudes (ranging from 20 to 385 m) within the country. This result corroborates well with the previous work of El-Niweiri and Moritz [35] in Sudan, who reported that the populations of *A. m. jemenitica* are scarce at altitudes below 500 m. 

The absence of honeybees from the M and C mtDNA lineages could be explained by the fact that these lineages are not endemic to West Africa [1,3]. In fact, the importation program of honeybees from the M lineage and Z sublineage initiated by the Government of Côte d’Ivoire in 1980 to improve the production of honey in the country had failed. The unsuccessful breeding of European honeybees in Côte d’Ivoire was due to the aggressiveness of the endemic honeybees against the imported honeybees and also to their poor adaptation to the new environment [36]. These factors significantly reduced the number of worker honeybees imported until the end of the honeybee importation program in Côte d’Ivoire in 1983. Therefore, the probability that these M and C lineages, and Z sublineage, were collected in our samples was very low.

The haplotype variants A_4_ occurred in the tropical dry climate in the ecological region of *A. m. scutellata* [35,36]. Haplotype A_1_ and its variants (A_1e_, A_1s_, A_1q_, and A_1r_) were recorded in the distributional area of *A. m. adansonii* [1,15,35]. The geographical distribution of the sequences recorded in this study is marked by a co-segregation of haplotypes and climate types. The genetic diversity of honeybee populations found in this study, therefore, might be a result of a long period of ecological adaptation to the environment. Thus, it is crucial to conserve the diversity of honeybee populations in Côte d’Ivoire.

## 5. Conclusions

According to our analyses of the intergenic *COI*-*COII* region of mtDNA, honeybee populations in Côte d’Ivoire exhibited the occurrence of two haplotypes, A_1_ and A_4_, with different variants i.e., A_1e_, A_1s_, A_1q_, A_1r_, A_4o_, and A_4r_. We did not find any local evidence of the introgression of imported honeybees from M and C lineages and Z sublineage. The haplotypes A_1_ and A_4_ occurred in the ecological regions of *A. m. adansonii* and *A. m. scutellata*, respectively. However, we were unable to fully infer the taxonomy of our honeybee colonies. Therefore, we recommend that future research should combine both morphometric and nuclear DNA analyses such as microsatellite markers for the accurate identification of the local honeybees in Côte d’Ivoire. Nevertheless, a larger sampling area is needed to be prospected to get a deeper insight into the biogeography of *A. mellifera* L. in Côte d’Ivoire.

## Figures and Tables

**Table 1:** Geo-coordinates, climatic conditions and landscape type of the sampling sites and colonies.

Climate Type	Total Number of Colonies (N)	Sampling Localities (Map Code)	Number of Colonies per Sampling Location	Latitude	Longitude	Altitude	Mean Annual Temperature (°C)	Mean Relative Humidity (%)	Mean Annual Precipitation (mm)	Biome Type
Tropical dry climate	10	Tengrela (1)	2	10°2′ N	6°42′ W	382	27.6	60.7	841	Savannah
		Ferkéssedougou (2)	2	9°6′ N	5°19′ W	370	27	68.0	951	
		Korhogo (3)	3	9°36′ N	5°63′ W	342	27	65.4	951	
		Tafiére (4)	3	8°4′ N	5°17′ W	385	26.6	65.0	1100	
Tropical humid climate	10	Didievi (5)	3	7°4′ N	4°3′ W	116	27.2	74.1	1090.6	
		Yamoussoukrou (6)	4	6°54′ N	5°21′ W	213	26	76.5	1118	
		Bouaflé (7)	2	6°59′ N	5°45′ W	205	26.6	75.8	1242	
		Duekoué (8)	1	6°45′ N	7°21′ W	234	25.4	78.2	1572	
Mountain climate	3	Man (9)	1	7°23′ N	7°31′ W	339	25	78	1930	Forest
		Danané (10)	2	7°16′ N	8°09′ W	354	24.9	77.2	1930	
Subequatorial climate	10	Dimbokro (11)	4	6°39′ N	4°42′ W	92	27	75	1176	
		Alepé (12)	1	5°3′ N	3°36′ W	89	26.5	82.7	1544	
		Dabou (13)	2	5°2′ N	4°23′ W	20	26.4	83.5	1743	
		Abobo (14)	1	5°25′ N	4°01′ W	108	26.5	82.7	1544	
		Bingerville (15)	2	5°21′ N	4°53′ W	59	26.5	90.4	1230	
